# Nationwide needs assessment on the potential use of virtual reality in teaching birth mechanics: perceptions of students and teaching professionals in midwifery and medicine in Germany

**DOI:** 10.1186/s12909-025-08532-6

**Published:** 2026-01-10

**Authors:** Kristina Vogel, Jana Adams, Rabi R. Datta, Nicola H. Bauer

**Affiliations:** 1 Institute of Midwifery Science, Faculty of Medicine and University Hospital Cologne, University of Cologne, Cologne, Germany; 2 Department of Obstetrics, Faculty of Medicine and University Hospital Cologne, University of Cologne, Cologne, Germany; 3 Department of General-, Visceral-, Thoracic- and Transplant Surgery,, Faculty of Medicine and University Hospital Cologne, University of Cologne, Cologne, Germany

**Keywords:** Extended reality, Midwifery education, Medical education, Virtual reality, Birth mechanics, Need assessment

## Abstract

**Background:**

A comprehensive understanding of birth mechanics is essential for safe and competent practice in obstetrics and midwifery. These dynamic, three-dimensional processes occur internally and are not directly observable, making them challenging to teach with traditional methods. Virtual Reality (VR) offers unique potential to visualize such complex, invisible mechanisms. This nationwide survey assessed the perceived need, curricular contexts, and preferred content for a high-fidelity VR application to teach birth mechanics in midwifery and medical education.

**Methods:**

An anonymous, digital survey was distributed to all study programs in midwifery science and medicine in Germany via program coordinators, department heads, and teaching leads in obstetrics and gynecology. Separate questionnaires for students and teaching professionals included 17 items on demographics, prior VR experience, attitudes toward VR, curricular timing, relevant learning objectives, and prioritized birth mechanics deviations. Responses were collected using Likert scales and multiple-choice questions, with non-parametric group comparisons (Mann–Whitney U, Chi-square test) and Bonferroni correction.

**Results:**

A total of 1,249 complete responses were analyzed with 22.4% (*n* = 280) teaching professionals and 77.6% (*n* = 969) students; 65.7% (*n* = 821) medicine, 34.3% (*n* = 428) midwifery. Overall, 76.3% reported a high or very high need for VR, and 90.2% held a positive or rather positive attitude toward its use. Midwifery teaching professionals reported more prior VR experience (56.7%) than medical ones (12.7%). Midwifery students favored early integration, while medical students preferred later, clinically oriented phases. The most frequently prioritized deviations in birth mechanics were occiput posterior position (73.3%), breech presentation (51.4%), and direct occiput position with high station (44.0%), with notable differences between disciplines and educational roles.

**Conclusion:**

This study reveals a strong perceived need for a VR application uniting anatomical accuracy with the dynamic processes of childbirth. Birth mechanics deviations were considered particularly suitable for simulation when they represent significant clinical challenges or are rarely demonstrated in current teaching. Tailored, interprofessional VR tools are perceived as potentially valuable for addressing these needs and may enhance obstetric and midwifery education. As part of the V.T.O.B.S. project (Virtual training for obstetric birth simulations), the next step will be the development and evaluation of such an application.

**Trial registration:**

The study protocol was preregistered in the German Clinical Trials Register (DRKS00035186 27.09.2024).

**Supplementary Information:**

The online version contains supplementary material available at 10.1186/s12909-025-08532-6.

## Background

A solid and sustainable understanding of birth mechanics is fundamental for both midwives and obstetricians to practice safely and confidently during childbirth. This knowledge enables healthcare professionals to distinguish physiological processes from pathological events and make appropriate decisions during childbirth. From an educational standpoint, teaching birth mechanics is crucial to help students develop the cognitive and practical skills needed in delivery room scenarios.

Birth mechanics involve the interaction of fetal and maternal anatomical structures in a dynamic, three-dimensional process. As this process occurs internally within the maternal body, it is not directly observable. Students must therefore mentally integrate anatomical knowledge of the pelvis and fetal head with a spatial understanding of fetal rotation and descent. This requirement for complex mental visualization, especially of processes that cannot be seen, often leads to cognitive overload [1]. Traditional teaching tools such as textbooks, static models, and 2D diagrams are limited in conveying these dynamic and spatial aspects. Consequently, learners frequently struggle to form accurate mental models, which can hinder comprehension [2], retention, and the practical application of knowledge in clinical settings [3]. These challenges make birth mechanics particularly well-suited to immersive simulation: they involve invisible, dynamic and spatially complex processes that are otherwise inaccessible to direct observation or conventional teaching tools.

From a theoretical perspective, the present study draws on concepts from technology acceptance research and cognitive learning theory to frame the constructs investigated. According to the Technology Acceptance Model (TAM) [4] and its extensions such as UTAUT [5], users’ acceptance of educational technology is shaped primarily by their perceived usefulness and attitudes toward the technology. These models have been widely applied in medical and health professions education to understand the adoption of digital and simulation-based tools. In addition, Cognitive Load Theory [6, 7] emphasizes the mental effort required to process complex visual-spatial information and the potential of interactive, multimodal environments to reduce extraneous load and foster deeper learning. Together, these frameworks informed the design of the present survey: the constructs of perceived need, attitude toward VR, and prioritized learning content were selected to capture key determinants of acceptance and perceived pedagogical value of immersive technologies in the context of teaching birth mechanics.

According to cognitive learning theories, immersive and interactive learning environments enhance the acquisition of complex and spatial knowledge [8, 9]. Research in medical education has demonstrated that 3D and VR-based simulations support the development of accurate mental models, reduce cognitive load, and increase learner motivation and engagement [10, 11]. These benefits apply particularly well to areas requiring spatial understanding, such as anatomy or surgical training [2, 11, 12]. Against this backdrop, the integration of modern teaching methods and innovative technologies has become increasingly vital in advancing medical and midwifery education, ensuring high-quality patient care and contributing to the continuous development of healthcare systems [13, 14]. Simulation-based learning has thus become an essential component of healthcare education, allowing students to apply theoretical knowledge in realistic, controlled settings and promoting the safe development of practical skills without putting patients at risk. While conventional simulation models, such as mannequins or standardized patients, offer value, they are often limited in their ability to depict internal, dynamic processes like those involved in childbirth. In contrast, immersive technologies such as Virtual Reality (VR) offers a distinct advantage. VR creates fully computer-generated environments experienced through head-mounted displays (HMDs) and interactive devices [15]. These simulations can replicate complex clinical scenarios with high fidelity, enabling learners to observe, engage with and rehearse real-world challenges. Importantly, they can visualize otherwise invisible mechanisms, such as the internal dynamics of birth mechanics, with high fidelity and interactivity. As such, VR is increasingly used in healthcare to enhance procedural training, clinical reasoning, teamwork and communication [16–19]. VR-based learning has been positively received in both medical and midwifery education. Learners report high satisfaction, emphasizing VR’s educational value and the benefit of a risk-free training environment [2, 20, 21].

Research has also shown improvements in confidence, motivation, and engagement, particularly in skill-based and emergency training scenarios [22]. For instance, VR simulations have improved learner performance in situations such as cesarean sections and premature rupture of membranes (PROM) [23]. XR and AR applications have shown positive impacts on preparedness, skill acquisition, and self-efficacy in obstetric emergency training [24].

The use of VR for teaching fetal lie, presentation, position, attitude and station in pregnancy did not result in a measurable knowledge gain [21, 25], although participants in both reported high satisfaction and self-confidence. These findings suggest that VR may still hold considerable potential in this area if integrated with pedagogically optimized content and assessment strategies.

Despite these encouraging indications, the overall body of research remains limited. Recent pilot studies have begun to investigate the potential of VR in midwifery education. Aasekjær et al. (2024) demonstrated that immersive VR can lead to sustained improvements in anatomical knowledge and spatial understanding of the female pelvic anatomy in a non-pregnant model [26]. Ljungblad et al. (2025) reported that mixed reality applications enhanced students’ comprehension of fetal rotation during labor, although the technology was primarily perceived as a supplement rather than a replacement for traditional teaching [27]. Ryan et al. (2022) found that VR applications focusing on fetal lie and presentation did not produce measurable knowledge gains, although students reported high satisfaction and increased confidence [21]. Other studies have taken broader or more specialized approaches. Baidoo et al. (2021) developed a comprehensive virtual birth scenario covering the entire intrapartum process from early labor to postpartum care, including dynamic maternal and fetal parameters and real-time clinical decision-making, with a primary emphasis on decision-making in complex clinical contexts rather than on the isolated visualization of birth mechanics [28]. In contrast, Hüseyinoğlu et al. (2025) focused on visualizing the three-dimensional relationship between fetal movements and maternal anatomy, aiming to improve students’ understanding of physiological processes that are otherwise hidden in clinical practice [29]. Collectively, these findings indicate that VR is perceived to promote motivation, engagement, and spatial understanding in midwifery education. However, existing research has been confined largely to midwifery students and has addressed either isolated aspects of anatomy, specific physiological processes, or broader clinical training environments. To date, no VR approach has comprehensively depicted the dynamic mechanics of childbirth in an interdisciplinary framework that also includes medical education.

Although recent initiatives, such as the PROGRESSION project or the study by Ryan et al. (2022), reflect growing interest in the use of VR for obstetric education, there remains a need for interactive, interdisciplinary simulations that present childbirth mechanics in real time with a multiprofessional educational perspective [21, 30].

The V.T.O.B.S. (Virtual training for obstetric birth simulations) project addresses this critical educational gap by developing a 360-degree immersive VR simulation that enables learners to explore the real-time dynamics of birth mechanics, with a focus on fetal rotation and maternal positioning. Designed from an interdisciplinary perspective, the simulation targets students and teaching professionals in both midwifery and medicine. In this context, virtual reality offers significant potential to support the acquisition of complex anatomical and physiological knowledge by enhancing the learning process and promoting a deeper, more sustainable understanding of childbirth mechanics. The overarching goal is to support sustainable learning through immersive visualization, helping students to build a robust understanding of the biomechanical processes involved in childbirth.

Based on the identified educational gap, this study seeks to answer the following research question: How can immersive Virtual Reality be used to effectively support the teaching of dynamic birth mechanics for students in midwifery and medicine?

To address this question, a nationwide needs assessment was conducted among students and teaching professionals in both midwifery and medicine. The study aims to systematically identify perceived educational needs, curricular contexts, and prioritized learning contents relevant for a future VR-based teaching tool on birth mechanics. By doing so, it provides a first empirical foundation for the pedagogical and technical development of an interdisciplinary VR application in obstetric education.

This approach fills a critical research gap: previous studies have either examined VR as an intervention to evaluate learning outcomes or focused on isolated aspects of anatomy or birth processes. In contrast, the present study adopts an exploratory, user-centered design to inform early-stage development and curricular integration. The methodological choice of an anonymous digital survey allows for large-scale participation across institutions, enabling a comprehensive overview of national trends and perceptions within the newly academicized field of midwifery education.

## Methods

This nationwide needs assessment was conducted within the framework of a larger project that ultimately led to the development of V.T.O.B.S (Virtual training for obstetric birth simulations), which was initiated to develop an immersive, interdisciplinary VR application that visualizes the dynamic mechanics of childbirth for midwifery and medical education.

### Ethical approval and registration

A digital and anonymous survey was created using *LimeSurvey Community Edition* (version 5.6.68 + 240625). Participation was voluntary. The study was approved by the Ethics Committee of the Medical Faculty of the University of Cologne on 7/12/2024 (ref number 24–1137). Additionally, the study protocol was preregistered in the German Clinical Trials Register (DRKS) under registration number DRKS00035186.

### Sample

Recruitment was conducted nationwide by contacting the respective department heads and teaching coordinators from the departments of gynecology and obstetrics, as well as the heads of midwifery institutes and study program coordinators across Germany via email. This approach targeted entire study programs in medicine and midwifery science, ensuring that both students and teaching professionals from all medical faculties and Universities of Applied Sciences offering these programs in Germany were reached.

#### Midwifery science programs

At the time of data collection, midwifery science was offered at 47 Universities and Universities of Applied Sciences across Germany. All 47 study program sites were contacted via email in October 2024. Study program coordinators were asked to forward the survey inventation to teaching professionals involved in academic instruction and, in a separate email, to distribute the survey link to enrolled students. Two reminder emails were sent (November 2024 and January 2025).

#### Medical programs 

At the time of data collection, undergraduate medical education in Germany was offered at 40 medical faculties. All medical faculties were contacted between October 2024 and January 2025. For the student survey, each faculty was contacted via publicly available institutional contacts. Depending on the locally accessible structures, invitations were addressed either to the student representative bodies (student councils) or to the dean’s or vice-dean’s offices for education, with the request to distribute the survey link to all enrolled medical students. For the teaching professional survey, teaching coordinators or educational leads of the university departments of obstetrics and gynecology were contacted at each faculty and asked to forward the survey link to all teaching staff involved in obstetrics and gynecology education. Two reminder emails were sent (November 2024 and January 2025).

It should be noted that student cohort sizes differ substantially between disciplines. In Germany, undergraduate medical education offers a comparatively large annual intake, with approximately 10,000–10,500 study places in medicine per year [31], as reported for recent admission cycles. In contrast, academic midwifery programs enroll markedly smaller cohorts nationwide. Following the academization of midwifery education, the annual number of available study places in midwifery science remains in the lower four-figure range, amounting to approximately 1,650 study places nationwide in 2024 [32], with relatively small cohorts per institution. These structural differences in annual study place capacity help explain the higher absolute number of responses from medical students compared to midwifery science and reflect differences in program size rather than recruitment strategy or engagement.

Because the survey was conducted fully anonymously and distributed via institutional intermediaries, and no information on institutional affiliation was collected to preserve anonymity, it was not possible to determine the exact number of students and teaching professionals who received the invitation or to calculate precise denominators and formal response rates for individual universities or subgroups. Accordingly, responses are reported descriptively based on the final sample.

A formal sample size calculation was not performed, as the study aimed for a comprehensive survey of all relevant teaching staff and students in Germany. The term *teaching professionals* was used throughout the study to uniformly refer to individuals involved in the academic instruction of birth-related content. This includes university-based teaching professionals from both obstetric/gynecological medicine and midwifery science. Specifically, it encompasses medical specialists (e.g., board-certified obstetricians/gynecologists), professors, research associates, and academic staff on the medical side, as well as lecturers without doctoral degrees on the midwifery side.

### Conceptual framework, survey development and structure

The conceptual design of the questionnaires was informed by established frameworks of technology acceptance (Technology Acceptance Model, TAM; Unified Theory of Acceptance and Use of Technology, UTAUT) and Cognitive Load Theory, which together emphasize perceived usefulness, attitudes toward technology, and cognitive demands as central determinants of technology adoption and learning effectiveness.

Two digital questionnaires were exploratively developed for this study to assess the perceived potential of VR for visualizing birth mechanics in medical and midwifery education, one for teaching professionals and one for students. Both instruments shared the same conceptual framework and comprised 17 items and an optional open-ended feedback section. The overarching aim was to capture perceived educational need, attitudes toward VR-based teaching, preferred curricular timing and context, and prioritized learning content related to birth mechanics. The items were derived from existing teaching objectives in obstetrics and midwifery curricula and developed collaboratively within the interdisciplinary project team to reflect both disciplinary expertise and educational relevance. The item pool was informed by established learning objectives commonly used in midwifery and medical education, as well as by discussions within the project team to ensure disciplinary accuracy and educational relevance.

As the study was designed as a nationwide needs assessment rather than a test of predefined latent constructs, the questionnaires were conceived as exploratory instruments focusing on descriptive insights to inform subsequent VR application design.

The questionnaire consisted of three thematic sections. “Sect. 1” (prior exposure and attitudes) addressed prior exposure to VR technology, general attitudes toward digital teaching, and the perceived usefulness of VR-based 360° visualization for understanding birth mechanics (e.g., “How do you assess the need to visualize the mechanics of childbirth using a VR 360° video?”). “Sect. 2” (professional background and curricular preferences) gathered professional background information (e.g., discipline, teaching role, and setting) and explored preferences for curricular integration, including appropriate semester and context for potential VR use. “Sect. 3” (learning objectives and prioritized deviations in birth mechanics) focused on learning content: participants rated predefined learning objectives for students (e.g., understanding birth mechanics, recognizing and managing anomalies in fetal position, posture, or presentation) and selected up to three deviations in birth mechanics they considered most relevant for VR-based depiction. An overview of the questionnaire structure and example items is provided in Table 1, and the full item wordings and response formats are available in English translation in supplementary files 1 and 2. These files include the full item wording, response formats, and section structure (Sects. 1–3) corresponding to the analyses reported in the Results.

**Table 1:** Overview of questionnaire sections and example items*

Section	Content focus	Example items	Response format
‍1	Prior exposure and attitudes toward VR	„How do you assess the need to visualize the mechanics of childbirth using VR 360° video?	‍5-point Likert: very low low moderate/medium high very high
‍2	Professional background and curricular preferences	„In which semester do you consider implementation to be appropriate? “	‍Multiple choice (semester selection)
‍‍‍‍3	Learning objectives and prioritized deviations in birth mechanics	„The students understand the anatomical fundamentals of the female pelvis and the birth process “	‍5-point Likert: strongly agree rather agree neither agree or disagree rather disagree strongly disagree

*Full versions of both questionnaires (teaching professionals and student) are available as Supplementary Files 1 and 2

Most items were assessed using five-point Likert scales. For example, participants rated the relevance of specific learning objectives related to birth mechanics on a scale from 1 (“strongly agree”) to 5 (“strongly disagree”).

Before data collection, both versions were reviewed by all members of the interdisciplinary project team to ensure clarity, content relevance, and consistency. Subsequently, a cognitive pre-test was conducted using the think-aloud method [33] with two participants from each target group (students and teaching professionals). During these sessions, participants verbalized their thoughts while completing the questionnaire. Feedback from this pilot phase was used to refine item wording, remove ambiguous formulations, align response options across items, and ensure that the intended meaning of each question was consistently understood. Minor adjustments were made to maximize content validity and face validity of the instrument for the purpose of a nationwide needs assessment.

### Data cleaning

A total of 1375 questionnaires were completed. To ensure data quality, cases with more than 20.0% missing values were excluded from the analysis (*n* = 126; 9.2%). This approach follows methodological guidance for case deletion when missing data are random [34]. A plausibility check revealed no systematic differences in demographic characteristics between included and excluded cases, suggesting data were missing at random. No formal sensitivity analyses were conducted beyond plausibility checks related to missing cases.

### Statistical analysis

Statistics were analyzed using IBM^®^ SPSS statistics (version 30.0). The Shapiro-Wilk test was used to assess normality. Since all variables significantly deviated from a normal distribution (*p* <.001), non-parametric methods were employed for group comparisons. The Mann-Whitney U test was used to analyze differences between the disciplines of medicine and midwifery science, as well as between students and teaching professionals. For categorical variables, such as the prioritization of birth mechanics deviations, the Chi-square test of independence (χ² test) was applied. For key proportions, 95% confidence intervals were calculated using Wilson score intervals. For the analysis of group differences in the prioritization of deviations in birth mechanics (nine individual Chi-square tests), a Bonferroni correction was applied to control for alpha inflation (adjusted significance level: α = 0.0056). Other analyses were interpreted using the conventional significance level of α = 0.05. Descriptive statistics (median, mean, standard deviation, interquartile range) were calculated to describe central tendencies and dispersion. Analyses were conducted by group, stratified by discipline (medicine, midwifery science) and educational role (students, educators). Effect sizes were interpreted following Cohen’s (1988) [35] conventions. *r* was calculated for Mann-Whitney U tests and *Cramer’s V* for Chi-square tests. For Chi-square analyses, expected cell counts were inspected. As some subgroup cells, particularly among teaching professionals, were small, results were interpreted cautiously and complemented by effect size report in (*Cramer’s V*), consistent with the exploratory nature of the study.

## Results

**Fig. 1:**
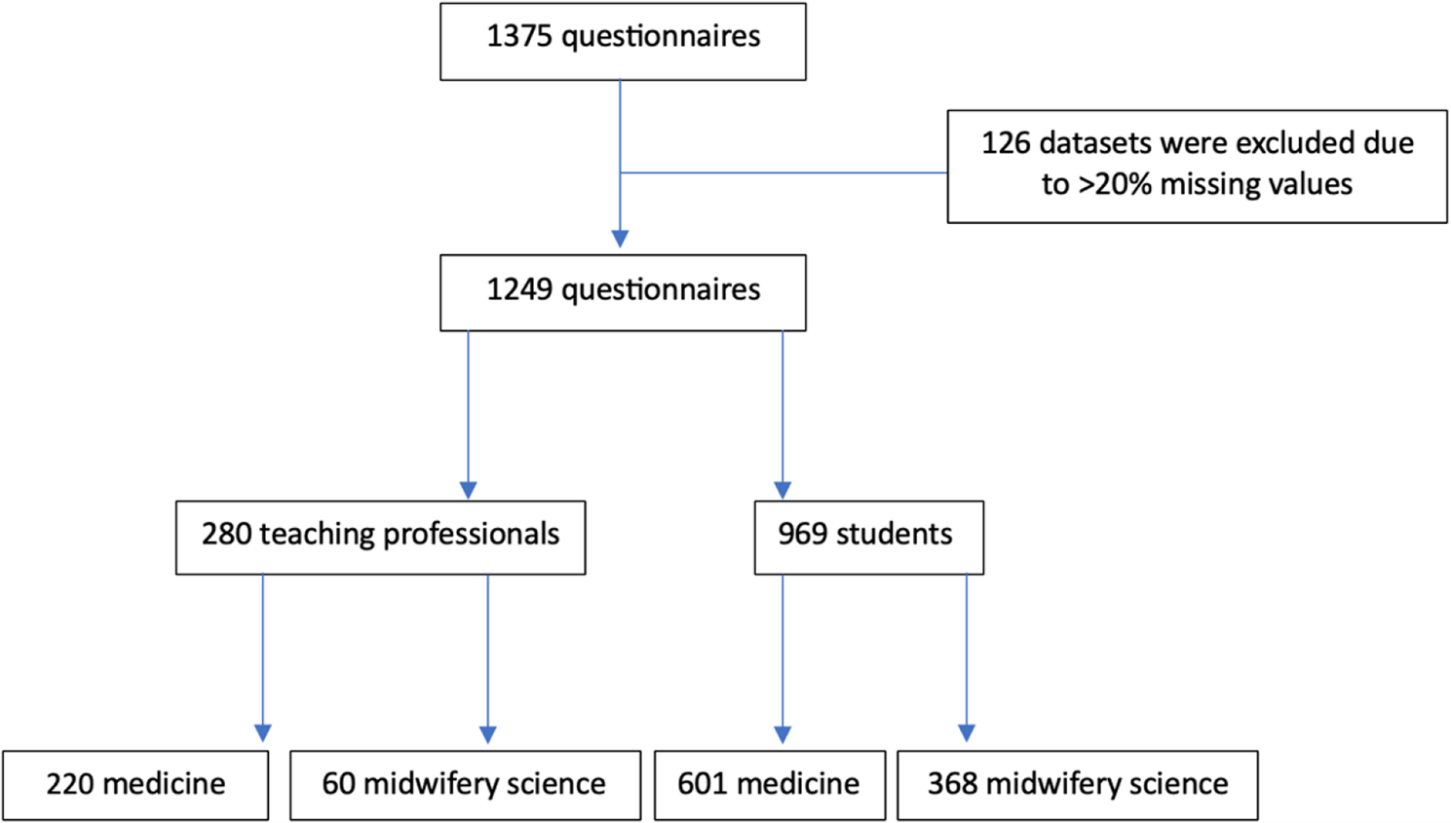
Composition of the datasets included in the final analysis

The final analysis included 1,249 complete datasets, subdivided into 280 (22.4%) teaching professionals and 969 students (77.6%). Of the teaching professionals, 220 (78.6%) were from medicine, while 60 (21.4%) work in midwifery science. Among the students, 601 (62.0%) are enrolled in medicine and 368 (38.0%) in midwifery science (Fig. 1).

The median semester of study for medical students was the 8th semester (IQR: 7–9; in a standard program of 10 semesters), while midwifery students were in the 3rd semester (IQR: 3–5; in a standard program of 6 to 8 semesters). The median age was 24 years (IQR: 22–25) for medical students and 22 years (IQR: 21–26) for midwifery students (Table 2).

**Table 2:** Participants demographic data

	Teaching professionals		Students	
	medicine (*n* = 220)	midwifery science (*n* = 60)	medicine (*n* = 601)	midwifery science (*n* = 368)
Age	n/a	n/a	24 [22–25]	22 [21–26]
Semester	n/a	n/a	8 [7–9]	3 [3–5]
Position				
Professor	n/a	16 (26.7)	n/a	n/a
Research associate	n/a	27 (45)	n/a	n/a
Lecturer without doctoral degrees	n/a	12 (20)	n/a	n/a
Other	n/a	5 (8.3)	n/a	n/a
VR experienced				
yes	28 (12.7)	34 (56.7)	217 (36.1)	153 (41.6)
no	192 (87.3)	26 (43.3)	384 (63.9)	213 (57.9)

*Age*: median, [IQR]

*Semester*: median, [IQR]

*Position* (midwives only): count, percentage (%)

*VR expectation*: count, percentage (%)

*n/a* not applicable

The majority of teaching professionals in midwifery science (96.7%; *n* = 58) reported independently designing courses for students. In addition, 80.0% (*n* = 48) regularly conducted practical exercises in the skills lab (midwifery teaching professionals only; survey instruments: Sect. 2; Items 2 C and 2D, Supp. 1).

Experience with VR varied between students and teaching professionals: While 56.7% (*n* = 34) of midwifery teaching professionals reported prior experience with VR, this was true for only 12.7% (*n* = 27) of medical teaching professionals. Among students, 41.6% (*n* = 153) of those studying midwifery and 36.1% (*n* = 217) of those studying medicine had previous experience with VR (survey instruments: Sect. 1; Item 1 A, Supp. 1–2).

### Attitudes toward and experience with VR

Approximately two-thirds of respondents (65.4%; *n* = 815) reported prior experience with VR, ranging from recreational use (e.g., rollercoaster simulations, gaming) to applications in academic courses, simulation training, and professional settings (survey instruments: Sect. 1; Item 1 A, Supp. 1–2). Attitudes toward the use of digital media in education were predominantly positive, with 92.9% (*n* = 1,159; survey instruments: Sect. 1; Item 1B, Supp. 1–2) rating its use as positive or rather positive. The perceived need for VR to visualize the mechanics of childbirth was rated as high or very high by 76.3% (*n* = 943) overall, and by 77.3% (*n* = 739) among students (survey instruments: Sect. 1; Item 1 C, Supp. 1–2). Similarly, the general attitude toward the use of VR for teaching birth mechanics was highly favorable, with 90.2% (*n* = 1,124) expressing positive or rather positive views (survey instruments: Sect. 1; Item 1D, Supp. 1–2).

### Timing and curricular contexts for VR use

Midwifery students expressed a preference for early integration of VR into the curriculum: 40.2% (*n* = 172) favored its use in the first semester, rising to 56.5% (*n* = 242) in the second and third semesters, followed by a steady decline in later stages (Student survey: Sect. 2; Item 2 C, Supp. 2). A large majority (92.1%; *n* = 394) considered VR a valuable tool to prepare for their first clinical placement, and 50.7% (*n* = 217) also for post-placement debriefing (Student survey: Sect. 2; Item 2D, Supp. 2).

Among medical students, the opposite trend was observed: agreement was low (< 1.5%) during the first four semesters, increasing markedly from the fifth semester onward, peaking in semesters seven (71.6%; *n* = 588) and eight (78.6%; *n* = 645) (Student survey: Sect. 2; Item 2 F, Supp. 2). Medical students predominantly endorsed VR for use in the clinical-practical phase, with 91.6% (*n* = 752) supporting its use during the clinical clerkship, 44.7% (*n* = 367) in elective rotations, 34.6% (*n* = 284) during the final practical year, and 19.2% (*n* = 158) during shorter clinical electives (Student survey: Sect. 2; Item 2G, Supp. 2). Overall, students in both disciplines associated the use of VR primarily with practice-oriented stages of their training, though midwifery students tended to favor earlier integration than medical students.

### Learning objectives and relevance of VR-based visualization of birth mechanics

Participants largely endorsed the predefined learning objectives for VR-based teaching. The strongest approval was given for understanding the anatomical fundamentals of the female pelvis and the birth process (74.3%; *n* = 924; “strongly agree”; survey instruments: Sect. 3, Item 3B, Supp. 1–2) and for comprehending the mechanism of labor in anterior occiput presentation (71.7%; *n* = 891, survey instruments, survey instruments: Sect. 3, Item 3 C, Supp. 1–2). Familiarity with the terminology of (non-physiological) birth mechanics was also widely supported (64.7%; *n* = 803; “strongly agree”, survey instruments: Sect. 3, Item 3 A, Supp. 1–2).

In contrast, objectives addressing more complex tasks, such as recognizing anomalies in fetal position, posture, or attitude (58.9%;*n* = 732 agreement, survey instruments: Sect. 3, Item 3D, Supp. 1–2) and applying management strategies for these deviations in birth mechanics (52.3%; *n* = 650; survey instruments: Sect. 3, Item 3E, Supp. 1–2), received lower yet consistently positive ratings. Linking vaginal examination findings to fetal presentation was also considered highly relevant (64.7%; *n* = 808; survey instruments: Sect. 3, Item 3 F, Supp. 1–2).

**Fig. 2:**
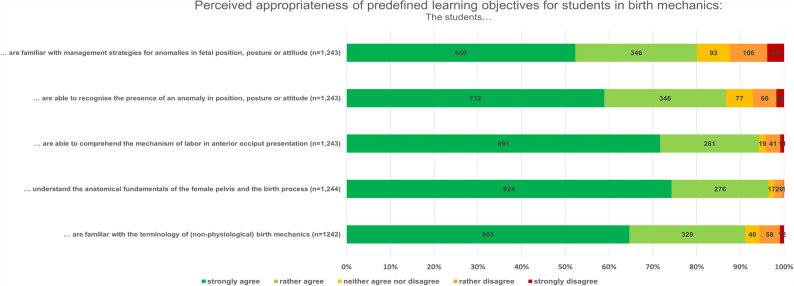
Perceived appropriateness of predefined learning objectives for students in birth mechanics (Items correspond to questionnaire Sect. 3: Learning Objectives). Participants (students and teaching professionals) rated the appropriateness of five predefined learning objectives formulated from the learner’s perspective (“the students…”) on a five-point Likert scale (strongly agree to strongly disagree). Bars represent the percentage distribution of responses

Overall, respondents viewed all proposed learning objectives as suitable for VR, with clearer consensus for foundational knowledge than for practical application skills (Fig. 2).

### Subgroup analysis: medicine vs. midwifery science

Medical participants more strongly agreed with the objective “are familiar with the terminology of (non-physiological) birth mechanics” (M = 1.47; Mdn = 1; survey instruments: Sect. 3; Item 3 A, Supp. 1–2) compared to those in midwifery science (M = 1.58; Mdn = 1, U = 153,900, Z = − 3.86, *p* <.001, *r* =.11). The same pattern was observed for “are able to recognize the presence of an anomaly in position, posture or attitude” (M = 1.59 vs. M = 1.71; U = 156,163; Z = − 3.31, *p* <.001, *r* =.10; survey instruments: Sect. 3; Item 3D, Supp. 1–2) and “are familiar with management strategies for anomalies in fetal position, posture or attitude ” (M = 1.78 vs. M = 1.95; U = 155,629; Z = − 3.28, *p* <.001, *r* = 0.09; survey instruments: Sect. 3; Item 3E, Supp. 1–2). No significant differences were found for “understanding anatomical fundamentals” (*p* =.421; survey instruments: Sect. 3; Item 3B, Supp. 1–2) or “following the course of occiput anterior delivery” (*p* =.756; survey instruments: Sect. 3; Item 3 C, Supp. 1–2). Effect sizes to Cohen’s classification indicated small to negligible effects across all comparisons.

### Subgroup analysis: students vs. teaching professionals

Teaching professionals rated “are able to comprehend the mechanism of labor in anterior occiput presentation” slightly higher (M = 1.29; Mdn = 1; survey instruments: Sect. 3; Item 3 C, Supp. 1–2) than students (M = 1.42; Mdn = 1, U = 123,977; Z = − 2.45, *p* =.014, *r* =.07). Students, in turn, rated “are able to recognize the presence of an anomaly in position, posture or attitude” (M = 1.61 vs. M = 1.70; U = 120,785; Z = − 2.95, *p* =.003, *r* =.08; survey instruments: Sect. 3; Item 3D, Supp. 1–2) and “are familiar with management strategies for anomalies in fetal position, posture or attitude” (M = 1.78 vs. M = 2.04; U = 108,133; Z = − 5.40, *p* <.001, *r* =.15; survey instruments: Sect. 3; Item 3E, Supp. 1–2) significantly higher than teaching professionals. No significant differences were found for “are familiar with the terminology of (non-) physiological birth mechanics” (*p* =.344; survey instruments: Sect. 3; Item 3 A, Supp. 1–2) or “understanding anatomical fundamentals” (*p* =.678; Sect. 3; Item 3B, Supp. 1–2). Although some group differences reached statistical significance, all corresponding effect sizes were small (*r* <.20), indicating limited practical relevance.

## Prioritization of deviations in birth mechanics for VR-b

ased depictionParticipants (*n* = 1,249) were asked to prioritize up to three deviations in birth mechanics, defined here as including anomalies in fetal position, posture, or attitude (survey instruments: Sect. 3; Item 3 H, Supp. 1–2). The most frequently mentioned were occiput posterior position (73.3%; *n* = 916), breech presentation (51.4%; *n* = 642), and direct occiput position with high station (44.0%; *n* = 549). Other common responses included brow presentation (34.5%; *n* = 431), parietal bone presentation (20.3%; *n* = 253), and deep transverse arrest (19.5%; *n* = 244). Less frequently prioritized were face presentation (12.6%; *n* = 157), sinciput presentation (10.6%; *n* = 133), and Roeder’s position (7.8%; *n* = 97).

### Subgroup analysis: prioritization of deviations in birth mechanics: midwifery students, medical students, midwifery teaching professionals, medical teaching professionals

For the subgroup comparison regarding prioritized deviations in birth mechanics (survey instruments: Sect. 3; Item 3 H), significance was evaluated using the Bonferroni-adjusted threshold (α = 0.0056). Under this criterion, several significant group differences remained (Table 3).

Overall, medical participants tended to prioritize deviations involving occiput positions and transverse arrests (e.g., occiput posterior position, direct occiput position with high station and deep transverse arrest), while midwifery participants more frequently selected complex fetal positions such as breech or parietal bone presentations.

Occiput posterior position was significantly more frequently prioritized by medical students (77.7%; *n* = 467) and teaching professionals (80.9%; *n* = 178) than by midwifery students (63.3%; *n* = 233) and teaching professionals (63.3%; *n* = 38), χ²(3) = 34.29, *p* <.001, *Cramer’s V* = 0.10.

Face presentation was significantly more often prioritized by midwifery students (18.5%; *n* = 68) than by medical students (10.5%; *n* = 63) or teaching professionals (medical: 9.5%; midwifery: 8.3%), χ²(3) = 16.88, *p* <.001, *Cramer’s V* = 0.12.

Parietal bone presentation showed large differences: 46.7% of midwifery teaching professionals selected it, compared to only 6.7% of medical students, χ²(3) = 160.69, *p* <.001, *Cramer’s V* = 0.36. Roeder’s position was also significantly more often chosen by midwifery students (14.7%; *n* = 54) and teaching professionals (15.0%; *n* = 9) than by medical students (2.0%; *n* = 12) and teaching professionals (10.0%; *n* = 22), χ²(3) = 58.36, *p* <.001, *Cramer’s V* = 0.22.

Direct occiput position with high station was most frequently prioritized by medical teaching professionals (64.5%; *n* = 142), χ²(3) = 49.00, *p* <.001, *Cramer’s V = 0.20*. Deep transverse arrest was particularly often selected by medical students (27.6%; *n* = 166) compared to midwifery teaching professionals (3.3%; *n* = 2), χ²(3) = 57.54, *p* <.001, *Cramer’s V* = 0.21.

Breech presentation was significantly more frequently chosen by midwifery students (62.5%; *n* = 230) and teaching professionals (66.7%; *n* = 40) than by medical students (55.4%; *n* = 333) and especially by medical teaching professionals (17.7%; *n* = 39), χ²(3) = 127.47, *p* <.001, *Cramer’s V* = 0.32.

Brow presentation and sinciput presentation showed no significant group differences after Bonferroni adjustment (Table 3). Corresponding 95% confidence intervals are provided in the Table 3.

Effect sizes (*Cramer’s V*) indicated mainly small to moderate associations between discipline or educational role and prioritization patterns, with the largest differences observed for parietal bone and breech presentation.

**Table 3:** Comparison of the prioritization of deviations in birth mechanics (including anomalies in fetal position, posture or attitude) among midwifery students, medical students, and teaching professionals in both fields

Birth mechanics deviations	Medical Students % (*n*) [95% CI]	Midwifery Students % (*n*) [95% CI]	Medical Teaching Professionals % (*n*) [95% CI]	Midwifery Teaching Professionals % (*n*) [95% CI]	χ²-value	df	*p*-value	Cramer’s V	Significance with Bonferroni (α = 0.0056)
Occiput posterior position	77.7 (467) [74.2–80.9]	63.3 (233) [58.3–68.1]	80.9 (178) [75.2–85.6]	63.3 (38) [50.7–74.4]	34.29	3	< 0.001	0.10	Sig.
Brow presentation	33.1 (199) [29.5–37.0]	31.5 (116) [27.0–36.4.0.4]	44.1 (97) [37.7–50.7]	31.7 (19) [21.3–44.2]	11.13	3	0.011	0.09	n.sig.
Sinciput presentation	9.0 (54) [7.0–11.5.0.5]	12.5 (46) [9.5–16.3]	12.7 (28) [9.0–17.8.0.8]	8.3 (5) [3.6–18.1]	4.41	3	0.220	0.06	n.sig.
Face presentation	10.5 (63) [8.3–13.2]	18.5 (68) [14.9–22.8]	9.5 (21) [6.3–14.2]	8.3 (5) [3.6–18.1]	16.88	3	< 0.001	0.12	Sig.
Parietal bone presentation	6.7 (40) [4.9–8.9]	37.2 (137) [32.4–42.3]	21.8 (48) [16.9–27.7]	46.7 (28) [34.6–59.1]	160.69	3	< 0.001	0.36	Sig.
Roeder’s Position	2.0 (12) [1.2–3.5]	14.7 (54) [11.42–18.7]	10.0 (22) [6.7–14.7]	15.0 (9) [8.1–26.1]	58.36	3	< 0.001	0.22	Sig.
Direct occiput position with high station	41.1 (247) [37.2–45.1]	36.1 (133) [31.4–41.2]	64.5 (142) [58.0–70.6.0.6]	45.0 (27) [33.1–57.5]	49.00	3	< 0.001	0.20	Sig.
Deep transverse arrest	27.6 (166) [24.2–31.3]	9.8 (36) [7.2–13.3]	18.2 (40) [13.7–23.8]	3.3 (2) [0.9–11.36.9.36]	57.54	3	< 0.001	0.21	Sig.
Breech presentation	55.4 (333) [51.4–59.3]	62.5 (230) [57.5–67.3]	17.7 (39) [13.3–23.3]	66.7 (40) [54.1–77.3]	127.47	3	< 0.001	0.32	Sig.

## Discussion

This nationwide needs assessment aimed to explore perceptions of students and teaching professionals in midwifery science and medicine regarding the potential use of VR to teach the complex topic of birth mechanics. While immersive technologies are increasingly integrated into health professions education, the majority of existing obstetric VR applications focus on emergency scenarios such as postpartum hemorrhage, shoulder dystocia, or cesarean section training [36, 37].

In this context, the present study contributes to the emerging body of evidence by mapping current perceptions and curricular needs as a prerequisite for designing and validating future VR interventions. It thereby occupies an early, exploratory position in the development cycle of educational technology research.

The consistently positive perceptions identified in this study align with technology acceptance models (TAM, UTAUT), which emphasize perceived usefulness and attitudes as central determinants of adoption. Accordingly, the high perceived educational value of VR indicates favorable conditions for curricular integration. Participants’ emphasis of visual and spatial aspects of birth mechanics resonates with principles of Cognitive Load Theory, highlighting the potential of interactive visualization to support mental model formation and reduce extraneous cognitive load.

Despite these promising theoretical underpinnings, most existing VR applications in obstetrics have visualized only isolated aspects of physiological birth [21, 27–29]. Few combine anatomically precise models with interactive, real-time representation of both physiological and non-physiological mechanisms across the full range of fetal positions, attitudes, lie, presentation, and station levels. An interdisciplinary perspective that addresses the needs of both midwifery and medicine education has been largely absent up to now. The evidence to date spans from knowledge gains to motivational benefits. Building on these findings, the present study extends existing work by identifying not only general acceptance but also the specific curricular and interprofessional conditions under which VR could be most effectively implemented.

Birth mechanics combine anatomical knowledge with dynamic, three-dimensional spatial reasoning, yet remain largely invisible during actual childbirth, making them challenging to grasp through traditional methods alone [1]. Prior work has shown that VR can both increase learner motivation and skills in complex intrapartum scenarios [28] and measurably improve understanding of physiological mechanisms such as the cardinal movements of labor [29]. This study is extending this line of research by identifying which aspects of physiological and non-physiological mechanisms members in both midwifery and medicine consider essential for VR-based teaching.

The present study is the first to systematically capture the needs of both professions in parallel, thereby providing an empirical foundation for developing an interdisciplinary VR application. Beyond this contribution to the emerging evidence on VR in obstetric and midwifery education, it also delivers empirically derived design requirements for a VR tool on birth mechanics, specifying curricular timing as well as the anatomical and functional features considered essential by students and teaching professionals. While Ryan et al. (2022) demonstrated that VR-based teaching of fetal orientation is well accepted but did not produce short-term knowledge gains [21], the present nationwide needs assessment extends this line of research by identifying which physiological and non-physiological mechanisms and which specific learning objectives should be prioritized, and how VR should be embedded into curricula. This provides an empirical foundation for the development of an interdisciplinary VR application that aims not only to foster motivation and confidence but also to support durable knowledge acquisition and application in practical contexts.

Understanding birth mechanics requires learners to mentally integrate pelvic anatomy, fetal head dimensions, and sequential rotations during descent - tasks associated with high cognitive load when using static models or 2D illustrations [1, 8]. Immersive VR has been shown to reduce cognitive load, enhance spatial understanding, and improve mental model accuracy, particularly in domains involving invisible or complex movements such as anatomy and surgical training [10–12, 38]. While existing digital resources, such as 3D animations or basic VR demonstrations, illustrate certain aspects of childbirth, they often lack the anatomical fidelity and interactive depth required for advanced learning. Our proposed application seeks to address these limitations by uniting high-resolution anatomical representation with the dynamic visualization of cardinal movements, maternal positioning, and selected deviations in fetal position and presentation. Beyond content, tackling design limitations is crucial to overcome known barriers such as usability concerns and side effects, which previous studies have identified as challenges for sustainable curricular integration [21, 27]. Such a tool holds particular value for interprofessional education: midwifery and medical students would train together on a shared, realistic depiction of both physiological and non-physiological processes, promoting a common language and mutual understanding across disciplines. This aligns with Cognitive Load Theory, which posits that interactive, multimodal representations can reduce extraneous load and enhance the construction of accurate mental models, particularly relevant when learning complex, dynamic spatial processes such as childbirth.

Consistently high levels of acceptance across all participant groups indicate favorable conditions for VR integration, particularly given the high proportions endorsing VR for visualizing birth mechanics and complex spatial processes [20, 21, 25].

While several studies emphasize VR as an educational supplement rather than a replacement [21, 27, 29] the Norwegian pilot study by Aasekjær et al. (2024) reported comparable or even superior results with VR alone, possibly reflecting increased learner autonomy [26]. This contrast highlights the importance of curricular framing. While Aasekjær et al. (2024) observed benefits when VR replaced lectures, the present needs assessment explicitly introduced VR as an add-on to ensure acceptance among students and teaching professionals.

However, experience levels differed markedly: teaching professionals in midwifery reported substantially more prior VR exposure than their medical counterparts. A likely contributing factor is the relatively recent academicization of midwifery education in Germany, with the first primary qualifying degree bachelor programs established in 2010 (BGBI. I 2009, p.3158) and full academicization mandated in 2020 (HebG 2019/2023). This has allowed curricula to be designed from the outset with modern simulation and digital tools, whereas medical curricula often evolve more incrementally. Similar discipline-based differences in technology adoption have been noted in simulation literature [16, 19].

Views on when VR should be integrated into the specific program differed: Midwifery students preferred to use it early, often in the first or second semester, to prepare for their practical training phases, whereas medical students saw greatest value in later stages with a stronger clinical focus. This suggests that curricular integration should be tailored to the learning trajectory of each study program. Aligning VR deployment with phases of maximal didactic benefit reflects established principles of simulation-based education [18] and is consistent with prior findings that the timing of immersive learning can influence both knowledge retention and learner engagement [26, 39]. Such tailored integration may be particularly important as interprofessional education gains increasing weight in health curricula. These discipline-specific patterns emphasize the need for flexible, modular integration of VR tools, consistent with the principles of constructive alignment, which call for synchronizing learning objectives, instructional methods, and assessment strategies across curricular stages. Participants strongly endorsed VR for teaching foundational anatomical concepts and normal childbirth progression, aligning with previous studies showing VR’s effectiveness in anatomy [26, 40, 41] and procedural skill development [37, 42]. Support was also high for more complex applications, such as recognizing deviations in fetal position, or presentation or selecting management strategies, though ratings were significantly lower than for core content. Medical teaching professionals and students placed greater emphasis on complex and differential aspects, whereas those in midwifery science prioritized core concepts. These discipline-specific preferences, also noted in other simulation research [21, 23], underline the value of a modular VR design that accommodates both foundational and advanced learning objectives. While several differences reached statistical significance, the corresponding effect size was small, suggesting that their practical relevance is limited. This modular approach also responds to the heterogeneity of prior findings, allowing VR to serve both as a motivational entry point and as a tool for knowledge-intensive learning.

Across groups, the most frequently prioritized deviations in birth mechanics for VR depiction were occiput posterior position, breech presentation, and direct occiput position with high station, each selected by a substantial proportion of respondents. These conditions combine high prevalence with elevated intervention risk, underscoring their clinical and educational relevance. Differences emerged between participant groups: students tended to prioritize management strategies, while teaching professionals focused more on basic mechanisms. This mirrors previous VR obstetric training research, where scenario choice balanced exposure to common presentations with uncommon scenarios that carry elevated clinical risk [30, 36]. Despite reaching statistical significance, these differences were associated with small effect sizes, suggesting that students and teaching professionals overall showed similar learning priorities. From a design perspective, sequencing modules from physiological to increasingly complex scenarios could accommodate both approaches. These results further guide the prioritization of educational content within an evidence-informed VR development framework, ensuring that simulation scenarios target clinically relevant and conceptually challenging processes.

The large number of respondents from both professions and educational roles underscores the high level of interest in immersive learning for birth mechanics. Beyond the descriptive scope, the findings offer strategic implications for interprofessional curriculum design. By identifying overlapping and distinct needs of medical and midwifery learners, the study highlights VR’s potential as a unifying educational medium that can foster shared understanding and collaborative competence across professions. Given VR’s proven utility for interprofessional simulation [17, 22], the findings of this nationwide needs assessment support the development of V.T.O.B.S. as a shared educational resource for midwifery and medicine. A modular architecture could allow tailoring of content depth, from core anatomy to advanced management of deviations in birth mechanics, matching learner experience and curricular phase. Additionally, by enabling visualization of otherwise inaccessible processes, such a tool could help standardize foundational knowledge across institutions, especially as midwifery programs continue to expand in Germany. Beyond Germany, these findings underscore the global relevance of VR for health professions education, as both midwifery and medical training worldwide face the challenge of integrating digital learning methods. By visualizing otherwise inaccessible processes, VR is perceived to hold particular promise for harmonizing the teaching of birth mechanics across professions. However, as these results are based solely on subjective perceptions, they should be interpreted as indicative of perceived educational needs and potentials rather than evidence of proven learning effects.

Taken together, these results contribute to the discourse on technology acceptance and immersive learning by highlighting how positive perceptions, curricular alignment, and interprofessional relevance support the sustainable integration of VR in health professions education.

### Limitations

This study has several limitations. First, although the survey aimed for comprehensive national coverage, participation was voluntary, which may have introduced a self-selection bias toward respondents with stronger interest in digital, immersive, or simulation-based learning. Second, all results are based on self-reported perceptions rather than objective measures of learning outcomes. The findings therefore reflect participants’ attitudes and perceived educational potential of VR, not demonstrated effects on knowledge, skills, or performance. Third, while the questionnaire was developed specifically for this exploratory study and underwent pretesting to ensure clarity and face validity, it was not psychometrically validated (e.g., factor analysis, internal consistency of multi-item scales). As the instrument was designed to capture a broad range of perceived needs, attitudes and content priorities rather than to generate composite scale scores, formal construct validation was not the primary focus of this work. Fourth, differences in group size, particularly between the large medical student cohort and smaller educator subgroups, may have influenced statistical comparisons. Moreover, as the study was conducted within the German educational context, transferability to other systems may be limited. Finally, the survey did not capture technical or infrastructural requirements, which will be crucial for implementation.

The next step, as part of the V.T.O.B.S. project, will be the iterative design and evaluation of the proposed VR application, incorporating the prioritized content and preferred curricular contexts identified here. First results from an undergraduate medical student cohort have already been published, showing high acceptance and feasibility, but not significant improvement in long-term OSCE performance after a single self-directed VR Session [43]. Further evaluations are still pending, including curricular implementation and effectiveness studies involving midwifery students. In this context, rigorous assessment should address not only user satisfaction and perceived utility but also knowledge retention, skill transfer, and clinical decision-making in both simulated and real environments.

Ultimately, such a tool has the potential to be integrated into standardized curricula nationwide, complementing existing simulation methods and contributing to the modernization of both medical and midwifery education. By combining high-fidelity anatomical accuracy with interactive, real-time dynamics, it may offer a transformative approach to teaching one of obstetrics’ most conceptually challenging yet fundamental topics.

## Conclusion 

This nationwide needs assessment identified a strong perceived need among midwifery and medical students and teaching professionals for an immersive VR application to teach birth mechanics. Respondents emphasized the value of VR for visualizing complex, internal processes that are otherwise inaccessible during real-life childbirth. In particular, deviations in birth mechanics were considered especially suitable for simulation when they represent relevant practical challenges or are rarely demonstrated effectively in current teaching. Preferences regarding timing, learning objectives, and the selection of deviations in birth mechanics varied between disciplines and educational roles, underlining the importance of tailoring immersive learning tools to curricular stage and professional focus. The high level of interest across groups underscores VR’s potential as a shared, interprofessional resource to bridge gaps in anatomy-based and dynamic birth process training. The next step, as part of the V.T.O.B.S project (Virtual training for obstetric birth simulations) will be the development and rigorous evaluation of a VR application that addresses these perceived needs, with the goal of empirically assessing its potential to enhance understanding, standardize teaching, and modernize the education of future midwives and obstetricians. It should be emphasized that these conclusions are based on participants’ reported perceptions and do not imply demonstrated learning efficacy, which will be the focus of subsequent evaluative research.

## Supplementary Information

Supplementary Material 1.

Supplementary Material 2.

## Data Availability

The datasets generated and analyzed during the current study are not publicly available as the project is still in progress and further analyses are planned. Data may be available from the corresponding author on reasonable request.

## References

[1] Badenhorst E, Mamede S, Hartman N, Schmidt HG. Exploring lecturers’ views of first-year health science students’ misconceptions in biomedical domains. Adv Health Sci Educ. 2015;20:403–20. 10.1007/s10459-014-9535-3. 10.1007/s10459-014-9535-3 25099944

[2] Moro C, Štromberga Z, Raikos A, Stirling A. The effectiveness of virtual and augmented reality in health sciences and medical anatomy. Anat Sci Educ. 2017;10:549–59. 10.1002/ase.1696. 28419750 10.1002/ase.1696

[3] Lufler RS, Zumwalt AC, Romney CA, Hoagland TM. Effect of visual–spatial ability on medical students’ performance in a gross anatomy course. Anat Sci Educ. 2011;5:3–9. 10.1002/ase.264. 22127919 10.1002/ase.264

[4] Davis FD. Perceived usefulness, perceived ease of use, and user acceptance of information technology. MIS Q. 1989;13:319–40. 10.2307/249008.

[5] Venkatesh V, Morris MG, Davis GB, Davis FD. User acceptance of information technology: toward a unified view. MIS Q. 2003;27:425–78. 10.2307/30036540.

[6] Sweller J. Cognitive load during problem solving: effects on learning. Cogn Sci. 1988;12:257–85. 10.1016/0364-0213(88)90023-7.

[7] Mayer RE. Cognitive theory of multimedia learning. In: Mayer RE, editor. The Cambridge handbook of multimedia learning. 2nd ed. Cambridge: Cambridge University Press; 2014. pp. 43–71. 10.1017/CBO9781139547369.005.

[8] Moreno R, Mayer RE. Learning science in virtual reality multimedia environments: role of methods and media. J Educ Psychol. 2002;94:598–610. 10.1037/0022-0663.94.3.598.

[9] Mayer RE, editor. The Cambridge handbook of multimedia learning. Cambridge University Press; 2014. 10.1017/CBO9781139547369.

[10] Neher AN, Bühlmann F, Müller M, Berendonk C, Sauter TC, Birrenbach T. Virtual reality for assessment in undergraduate nursing and medical education - a systematic review. BMC Med Educ. 2025;25:292. 10.1186/s12909-025-06867-8. 39987099 10.1186/s12909-025-06867-8 PMC11846274

[11] Mao RQ, Lan L, Kay J, Lohre R, Ayeni OR, Goel DP, et al. Immersive virtual reality for surgical training: A systematic review. J Surg Res. 2021;268:40–58. 10.1016/j.jss.2021.06.045. 34284320 10.1016/j.jss.2021.06.045

[12] Agbafe V, Jazayeri HE, Baker N, Cederna PS. Augmenting medical and surgical education with virtual reality. Plast Reconstr Surg. 2023;152:e556–8. 10.1097/PRS.0000000000010546. e. 10.1097/PRS.0000000000010546 37252919

[13] Kim H-Y, Kim E-Y. Effects of medical education program using virtual reality: A systematic review and Meta-Analysis. Int J Environ Res Public Health. 2023;20:3895. 10.3390/ijerph20053895. 36900904 10.3390/ijerph20053895 PMC10001289

[14] Neubacher M, Siebers P, Wittek A, Recker F. How to play a game Properly - Enhancing obstetrics and gynecology education through gamification: A scoping review. Geburtshilfe Frauenheilkd. 2024;84:1126–34. 10.1055/a-2379-8729. 39649126 10.1055/a-2379-8729 PMC11623999

[15] Izard SG, Juanes JA, García Peñalvo FJ, Estella JMG, Ledesma MJS, Ruisoto P. Virtual reality as an educational and training tool for medicine. J Med Syst. 2018;42:50. 10.1007/s10916-018-0900-2. 29392522 10.1007/s10916-018-0900-2

[16] Lendahls L, Oscarsson MG. Midwifery students’ experiences of simulation- and skills training. Nurse Educ Today. 2017;50:12–6. 10.1016/j.nedt.2016.12.005. 28006699 10.1016/j.nedt.2016.12.005

[17] Lindsay Miller J, Avery MD, Larson K, Woll A, VonAchen A, Mortenson A. Emergency birth hybrid simulation with standardized patients in midwifery education: implementation and evaluation. J Midwifery Womens Health. 2015;60:298–303. 10.1111/jmwh.12276. 25963413 10.1111/jmwh.12276

[18] Letterie GS. How virtual reality May enhance training in obstetrics and gynecology. Am J Obstet Gynecol. 2002;187:37–40. 10.1067/mob.2002.127361 12235439

[19] Changuiti O, Marfak A, Saad E, Hilali A, Benjouad A, Youlyouz-Marfak I. Simulation and midwifery education 2011–2021: a systematic review. Br J Midwifery. 2023;31:286–93. 10.12968/bjom.2023.31.5.286.

[20] Tursø-Finnich T, Jensen RO, Jensen LX, Konge L, Thinggaard E. Virtual reality Head-Mounted displays in medical education. Simul Healthcare: J Soc Simul Healthc. 2023;18:42–50. 10.1097/SIH.0000000000000636. 10.1097/SIH.0000000000000636 35136005

[21] Ryan G, Callaghan S, Rafferty A, Murphy J, Higgins M, Barry T, et al. Virtual reality in midwifery education: A mixed methods study to assess learning and Understanding. Nurse Educ Today. 2022;119:105573. 10.1016/j.nedt.2022.105573. 36206631 10.1016/j.nedt.2022.105573

[22] McKelvin R, McKelvin G. Immersive simulation training: comparing the impact on midwifery and paramedic students’ confidence to perform basic life support skills. Midwifery. 2020;87:102717. 10.1016/j.midw.2020.102717. 32353663 10.1016/j.midw.2020.102717

[23] Kim HJ, Lee HK, Jang JY, Lee K-N, Suh DH, Kong H-J, et al. Immersive virtual reality simulation training for Cesarean section: a randomized controlled trial. Int J Surg. 2023. 10.1097/JS9.0000000000000843. 10.1097/JS9.0000000000000843 PMC10793750 37939117

[24] Vogel K, Bernloehr A, Willmeroth T, Blattgerste J, Hellmers C, Bauer NH. Augmented reality simulation-based training for midwifery students and its impact on perceived knowledge, confidence and skills for managing critical incidents. Midwifery. 2024;136:104064. 10.1016/j.midw.2024.104064. 38905862 10.1016/j.midw.2024.104064

[25] Aydın Doğan R, Yazıcı S. Use and effectiveness of innovative virtual reality application in teaching fetal development: A randomized controlled trial. Comput Inf Nurs. 2024;42:515–21. 10.1097/CIN.0000000000001036. 10.1097/CIN.0000000000001036 38453431

[26] Aasekjær K, Bjørnås B, Skivenes HK, Vik ES. Immersive virtual reality (VR) when learning anatomy in midwifery education: a pre-post pilot study. Eur J Midwifery. 2024;8:1–7. 10.18332/ejm/191364. 10.18332/ejm/191364 PMC11350978 39206326

[27] Ljungblad LW, Murphy D, Fonkalsrud HE. Mixed reality for midwifery students: a qualitative study of the technology’s perceived appropriateness in the classroom. BMC Med Educ. 2025;25:337. 10.1186/s12909-025-06919-z. 40038660 10.1186/s12909-025-06919-z PMC11881337

[28] Baidoo K, Adu C. Virtual reality training to enhance clinical competence and student engagement in Ghana. Br J Midwifery. 2025;33:6–12. 10.12968/bjom.2024.0037.

[29] Hüseyinoğlu S, Yazıcı S. The use and efficacy of an innovative virtual reality application in teaching the mechanism of labor: A randomized controlled trial. Clin Simul Nurs. 2025;100:101698. 10.1016/j.ecns.2025.101698.

[30] ProgressionDeep understanding of positioning for midwives in obstetrics using modern technologies (AR/VR).Available from: https://progression.inm-online.de/. Accessed 2024 Aug 26. } } https://progression.inm-online.de/}

[31] CHE – Centrum für Hochschulentwicklung. Medizinstudienplätze in den deutschen Bundesländern. CHE Hochschuldaten; 2025 Aug 20. Available from:https://hochschuldaten.che.de/medizinstudienplaetze-in-den-deutschen-bundeslaendern/. Accessed 2025 Dec 17 https://hochschuldaten.che.de/medizinstudienplaetze-in-den-deutschen-bundeslaendern/}

[32] Deutscher Hebammenverband e. V. Entwicklung der Ausbildungszahlen von Hebammen von der Ausbildung an Hebammenschulen zum primärqualifizierenden Studiengang Hebammenwissenschaft an Hochschulen und Universitäten . Berlin: Deutscher Hebammenverband e. V.; 2025 Feb 12. } Available from:https://hebammenverband.de/wp-content/uploads/2025/02/2025_02_12_Ausbildungszahlen-Hebammen.pdf. Accessed 2025 Dec 17 Clear } } https://hebammenverband.de/wp-content/uploads/2025/02/2025_02_12_Ausbildungszahlen-Hebammen.pdf}

[33] Ericsson KA, Simon HA. How to study thinking in everyday life: contrasting think-aloud protocols with descriptions and explanations of thinking. Mind Cult Act. 1998;5:178–86. 10.1207/s15327884mca0503_3.

[34] Tabachnick BG, Fidell LS. Using multivariate statistics. 6th ed. Pearson International; 2013. https://elibrary.pearson.de/book/99.150005/9781292034546.

[35] Cohen J. Statistical power analysis for the behavioral sciences. 2nd ed. Hillsdale, NJ: Lawrence Erlbaum; 1988.

[36] Falcone V, Catic A, Heinzl F, Steinbauer P, Wagner M, Mikula F, et al. Impact of a virtual reality-based simulation training for shoulder dystocia on human and technical skills among caregivers: a randomized-controlled trial. Sci Rep. 2024;14:7898. 10.1038/s41598-024-57785-6. 38570525 10.1038/s41598-024-57785-6 PMC10991516

[37] McEvoy A, Kane D, Hokey E, Mangina E, Higgins S, McAuliffe FM. Virtual reality training for postpartum uterine balloon insertion-a multi-center randomized controlled trial. Am J Obstet Gynecol MFM. 2024;6:101429. 10.1016/j.ajogmf.2024.101429. 39019213 10.1016/j.ajogmf.2024.101429

[38] Moro C, Phelps C, Redmond P, Stromberga Z. HoloLens and mobile augmented reality in medical and health science education: A randomised controlled trial. Br J Edu Technol. 2021;52:680–94. 10.1111/bjet.13049.

[39] Gosavi A, Kanneganti A, Khoo ET, Singh K, Shen L, Rauff M, et al. Virtual reality simulation training for childbirth: A cluster randomized crossover study. Int J Gynecol Obstet. 2025;170:681–90. 10.1002/ijgo.70053. 10.1002/ijgo.70053 PMC12255915 40156490

[40] Ellington DR, Shum PC, Dennis EA, Willis HL, Szychowski JM, Richter HE. Female pelvic floor immersive simulation: A randomized trial to test the effectiveness of a virtual reality anatomic model on resident knowledge of female pelvic anatomy. J Minim Invasive Gynecol. 2019;26:897–901. 10.1016/j.jmig.2018.09.003. 30218709 10.1016/j.jmig.2018.09.003

[41] Ryan G, Rafferty A, Murphy J, Higgins MF, Mangina E, McAuliffe FM. Virtual reality learning: A randomized controlled trial assessing medical student knowledge of fetal development. Int J Gynecol Obstet. 2023;162:292–9. 10.1002/ijgo.14684. 10.1002/ijgo.14684 36883288

[42] Kane D, Ryan G, Mangina E, McAuliffe FM. A randomized control trial of a virtual reality learning environment in obstetric medical student teaching. Int J Med Inf. 2022;168:104899. 10.1016/j.ijmedinf.2022.104899. 10.1016/j.ijmedinf.2022.104899 36335797

[43] Adams J, Klein C, Ludwig S, Stosch C, Vogel K, Bauer NH, Bruns CJ, Datta RR. V.T.O.B.S.—Learning birth mechanics in virtual reality: a controlled cohort study in undergraduate medical education. Front Med. 2025;12:1715561. 10.3389/fmed.2025.1715561. 10.3389/fmed.2025.1715561 PMC12647056 41312483

