# Supplementary material for: Nationwide needs assessment on the potential use of virtual reality in teaching birth mechanics: perceptions of students and teaching professionals in midwifery and medicine in Germany
Source: BMC Med Educ. 2026 Jan 10;26:146. doi: 10.1186/s12909-025-08532-6 (PMC12849493; doi:10.1186/s12909-025-08532-6)
Supplement: Supplementary file 1 — Supplementary Material 1. [file 12909_2025_8532_MOESM1_ESM.pdf]

Universität zu Köln
Medizinische Fakultät

Survey on the Pontential Use of Virtual Reality (VR) in Teaching Birth Mechanics: Teaching Professionals

Dear teaching professionals,

We (the research team for obstetrics and midwifery science at the Faculty of Medicine at the University of Cologne) are interested in your experience and opinion. We would like to support students of medicine and midwifery science in better understanding the specifics of birth mechanics. Understanding birth mechanics and supporting the birth process are relevant parameters in birth care that should be mastered by obstetricians and midwives. To this end, we are planning to develop an interactive 360-degree video using virtual reality (VR). The VR simulation is to be used with VR glasses. A woman in labor (in different birthing positions) is depicted. It is possible to look directly into the body of the woman in labor, so that the fetus can be seen in different positions (location, position, posture, pole position, position) in the female pelvis can be visualized and the process of entry into the pelvic inlet, the passage mechanism, the exit mechanism, and the external rotation of the head and shoulder delivery can be displayed in the form of a video animation (in 3D). The video animation can be stopped at any time, and the user can also change the perspective to get a more detailed view of what is happening.

Important note: VR simulation does not replace seminars or lectures. VR simulation should be understood as an addon and is intended to offer the opportunity to deepen existing knowledge and help develop an understanding of the mechanics of childbirth.

[1]Definition of virtual reality (VR): The user is completely immersed in a computer-generated 360-degree world with interactive possibilities through VR glasses. No content from the real world is retained in the simulation.

Instructions for completing the questionnaire

- Your questionnaire and your information will be treated anonymously: This means that no one will be able to link you to your questionnaire or your answers afterwards. **Participation in the study is voluntary. If you do not wish to participate in the study, there will of course be no disadvantages for you in any way.**
- Completing the entire questionnaire takes approximately 10 minutes.
- Please **read** through **all the possible answers** before choosing one or, in the case of multiple answers, several.
- Please **answer every question** or **statement**.
- If you have any questions or comments, please feel free to contact us at any time.

We would ask you to share the survey with your colleagues, especially those who are involved in teaching.
Thank you for your participation!
Sincerely, Dr. Jana Adams, Kristina Vogel, M.Sc., Univ.-Prof.in Dr. Nicola H. Bauer & Priv.-Doz. Dr. Rabi Datta

1 Virtual Reality

1A Have you already been in contact with VR?

No ☐	Yes ☐ In the following context (please specify): ______ ______

1B What is your overall opinion of the use of digital media in the classroom?

Positive	☐
Rather positive	☐
Neutral	☐
Rather negative	☐
Negative	☐

1C How do you assess the need among students to visualize the mechanics of childbirth using VR 360° video?

Very low	☐
Low	☐
Moderate/medium	☐
High	☐
Very high	☐

1D What is your attitude toward using Virtual Reality (VR) to learn about and explore the topic of birth mechanics?

Positive	☐
Rather positive	☐
Neutral	☐
Rather skeptical	☐
Skeptical	☐

Universität zu Köln
Medizinische Fakultät

2 Profession

2A Which discipline do you belong to?	
Medicine	☐ ➔ **Please continue with question 2 G**
Midwifery science	☐

2B What is your professional position?)→ midwifery only	
Professor	☐
Research assistant	☐
Lecturer without doctoral degrees	☐
Other: ____________________	☐

2C Do you independently teach courses for midwifery students?)→ midwifery science only				
Yes	☐	No	☐	

2D Do you independently teach courses in the skills lab for midwifery students?)→ midwifery science only				
Yes	☐	No	☐	

2E In which semester do you consider implementation to be appropriate (multiple answers possible)→ midwifery science only					
1	☐	2	☐	3	☐
4	☐	5	☐	6	☐
7	☐	8	☐		

2F In which context do you consider the use to be appropriate? (Multiple answers and free text additions possible)	
Before clinical practice (as preparation)	☐
After clinical practice (as follow-up)	☐
Other: ____________	☐
➔ **Please continue with section 3 Learning objectives**	

2G In which semester do you consider implementation to be appropriate (multiple answers possible)→ Medicine only					
1	☐	2	☐	3	☐
4	☐	5	☐	6	☐
7	☐	8	☐	9	☐
10	☐	Final year (practical)	☐		

2H In which context do you consider the use to be appropriate? (Multiple answers and free-text additions possible) → Medicine only	
Clinical rotation (all students)	☐
Elective module/course	☐
Clinical traineeship (Famulatur)	☐
Final year (practical)	☐
Other: ________________________________	☐

Universität zu Köln
Medizinische Fakultät

3 Learning objectives

The following learning objectives were defined in advance by the project team. To what extent do you agree that these objectives are appropriate?
The students...

3A ...are familiar with the terminology of (non-physiological) birth mechanics	
Strongly agree	☐
Rather agree	☐
Neither agree nor disagree	☐
Rather disagree	☐
Strongly disagree	☐

3B... understand the anatomical fundamentals of the female pelvis and the birth process	
Strongly agree	☐
Rather agree	☐
Neither agree nor disagree	☐
Rather disagree	☐
Strongly disagree	☐

3C… are able to comprehend the mechanism of labor in anterior occiput presentation	
Strongly agree	☐
Rather agree	☐
Neither agree nor disagree	☐
Rather disagree	☐
Strongly disagree	☐

3D... are able to recognise the presence of an anomaly in position, posture or attitude	
Strongly agree	☐
Rather agree	☐
Neither agree nor disagree	☐
Rather disagree	☐
Strongly disagree	☐

Universität zu Köln
Medizinische Fakultät

3E... are familiar with management strategies for anomalies in fetal position, posture or attitude	
Strongly agree	☐
Rather agree	☐
Neither agree nor disagree	☐
Rather disagree	☐
Strongly disagree	☐

3F Which learning objectives, in your opinion, would be relevant in addition to the ones mentioned above? (Free-text)

3G How relevant do you consider the visualization of a vaginal finding and its subsequent connection to the fetal position for the students?	
Very relevant	☐
Relevant	☐
Neutral	☐
Not relevant	☐
Not relevant at all	☐

3H Which birth mechanics deviations would you prioritize for implementation in VR? (Please select up to three.)					
Occiput posterior position	☐	Brow presentation	☐	Sinciput presentation	☐
Face presentation	☐	Parietal bone presentation	☐	Roeder's Position	☐
Direct occiput position with high station	☐	Deep transverse arrest	☐	Breech presentation	☐

4 Feedback

Do you have any additional comments? What else do you think might be relevant for implementation?

We sincerely thank for your participation!
